# Supplementary material for: Causal Relationship between Aging and Anorexia Nervosa: A White-Matter-Microstructure-Mediated Mendelian Randomization Analysis
Source: Biomedicines. 2024 Aug 16;12(8):1874. doi: 10.3390/biomedicines12081874 (PMC11351342; doi:10.3390/biomedicines12081874)
Supplement: Supplementary file 1 [file biomedicines-12-01874-s001.zip › Supplementary Figure/Figure S4/Singe SNP plot of MR analysis for TL to ubm-b-1737.pdf]

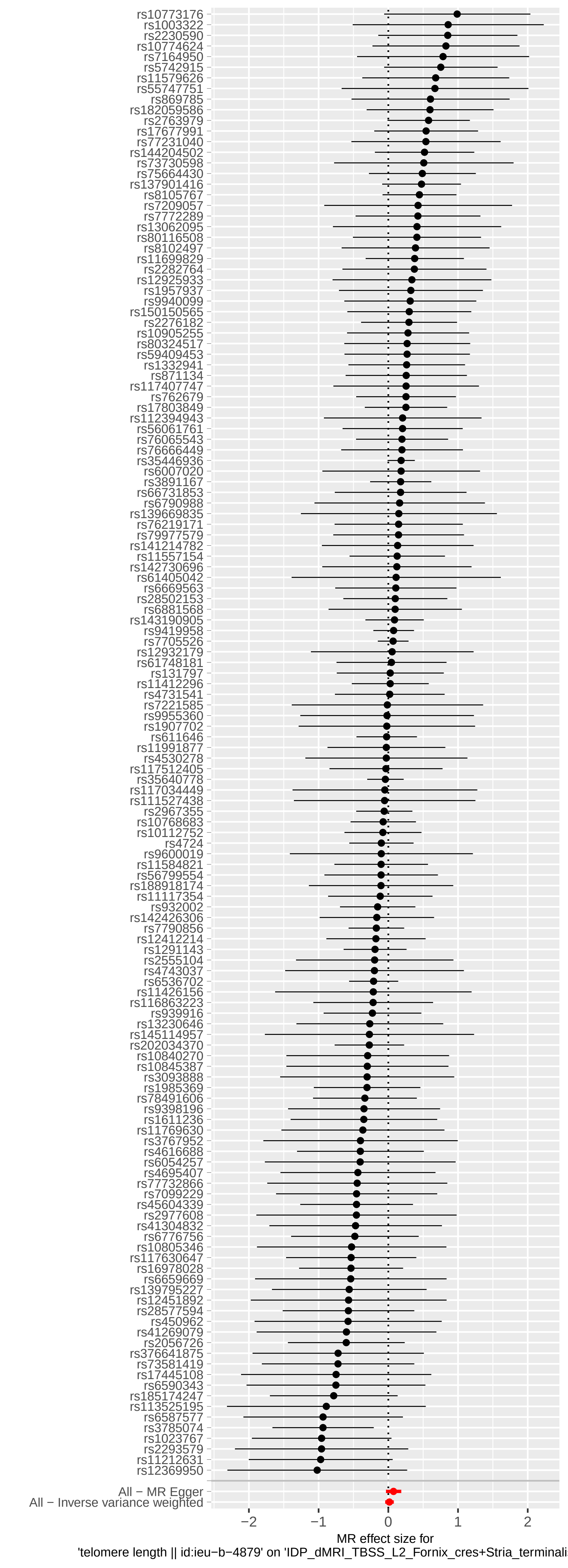

rs10773176
rs1003322
rs2230590
rs10774624
rs7164950
rs5742915
rs11579626
rs55747751
rs869785
rs182059586
rs2763979
rs17677991
rs77231040
rs144204502
rs73730598
rs75664430
rs137901416
rs8105767
rs7209057
rs7772289
rs13062095
rs80116508
rs8102497
rs11699829
rs2282764
rs12925933
rs1957937
rs9940099
rs150150565
rs2276182
rs10905255
rs80324517
rs59409453
rs1332941
rs871134
rs117407747
rs762679
rs17803849
rs112394943
rs56061761
rs76065543
rs76666449
rs35446936
rs6007020
rs3891167
rs66731853
rs6790988
rs139669835
rs76219171
rs79977579
rs141214782
rs11557154
rs142730696
rs61405042
rs6669563
rs28502153
rs6881568
rs143190905
rs9419958
rs7705526
rs12932179
rs61748181
rs131797
rs11412296
rs4731541
rs7221585
rs9955360
rs1907702
rs611646
rs11991877
rs4530278
rs117512405
rs35640778
rs117034449
rs111527438
rs2967355
rs10768683
rs10112752
rs4724
rs9600019
rs11584821
rs56799554
rs188918174
rs11117354
rs932002
rs142426306
rs7790856
rs12412214
rs1291143
rs2555104
rs4743037
rs6536702
rs11426156
rs116863223
rs939916
rs13230646
rs145114957
rs202034370
rs10840270
rs10845387
rs3093888
rs1985369
rs78491606
rs9398196
rs1611236
rs11769630
rs3767952
rs4616688
rs6054257
rs4695407
rs77732866
rs7099229
rs45604339
rs2977608
rs41304832
rs6776756
rs10805346
rs117630647
rs16978028
rs6659669
rs139795227
rs12451892
rs28577594
rs450962
rs41269079
rs2056726
rs376641875
rs73581419
rs17445108
rs6590343
rs185174247
rs113525195
rs6587577
rs3785074
rs1023767
rs2293579
rs11212631
rs12369950
All − MR Egger
All − Inverse variance weighted
−2
−1
0
1
2
MR effect size for
'telomere length || id:ieu−b−4879' on 'IDP_dMRI_TBSS_L2_Fornix_cres+Stria_terminali
